# Anti-N-Methyl-D-Aspartate Receptor Encephalitis Complicated by Autoimmune Enteropathy and Pulmonary Embolism: A Rare Case

**DOI:** 10.7759/cureus.26496

**Published:** 2022-07-01

**Authors:** Maryam B Haider, Noren Din, Sophia Dar, Brinda Basida

**Affiliations:** 1 Internal Medicine, Detroit Medical Center/Wayne State University, Sinai-Grace Hospital, Detroit, USA; 2 Internal Medicine, Hackensack University Medical Center, Hackensack, USA; 3 Internal Medicine, Detroit Medical Center Sinai-Grace Hospital, Detroit, USA

**Keywords:** gut-brain axis, autonomic enteropathy, pulmonary embolism, pseudo-obstruction, n-methyl-d-aspartate (nmda) encephalitis

## Abstract

Anti-N-methyl-D-aspartate (anti-NMDA) receptor encephalitis is an autoimmune disorder affecting the N-methyl-D-aspartate receptors in the central and peripheral nervous systems. Gastrointestinal (GI) complications are rarely manifested in this disease. Autoimmune dysregulation of the GI tract is considered a potential cause. We present a challenging case of a 38-year-old male with a history of newly diagnosed epilepsy. He was admitted for three weeks of confusion, hallucinations, and bizarre behavior, and was later diagnosed with anti-NMDA encephalitis from a cerebrospinal fluid (CSF) immunological study. He was treated with a five days course of intravenous immunoglobulin (IVIG) and high-dose steroids. His course was further complicated with GI obstruction and upper GI bleed. His laboratory workup showed lactic acidosis and there was a concern for ischemic bowel injury. Computed tomography (CT) of the abdomen with contrast showed diffuse moderate to pronounced dilated small intestine swirling the mesenteric vessels, concerning for intestinal vascular compromise. The patient also became tachypneic and hypoxic, requiring 6 L of oxygen with a venti-mask. CT of the chest, abdomen, and pelvis with contrast revealed saddle pulmonary embolism (PE) extending to the right and left pulmonary arteries with right heart strain. He underwent emergent explorative laparotomy and emergent catheter-directed thrombectomy. Neither necrotic bowel nor any evidence of perforation or volvulus was noted during the laparotomy; however, the small bowel and the colon were reported to be significantly dilated, hyperemic, and engorged with blood without any evidence of ischemic bowel. He had a complicated 29-day admission course and recovered functional capacity to be safely discharged to a skilled nursing facility for further care. Physicians should keep in mind the gut-brain axis and autonomic effects on gut receptors of any patient presenting with psychosis and seizure disorder to provide timely care and improve morbidity and mortality in this patient population.

## Introduction

N-methyl-D-aspartate (NMDA) encephalitis is autoimmune encephalitis characterized by various antineuronal autoantibodies to the NMDA receptor (NMDAR) involving the limbic structures, neocortex, hindbrain, striatum, spinal cord, and peripheral nervous system [1]. Clinical presentation varies drastically from psychosis and hallucination to altered mental status. NMDA encephalitis is a sporadic disease affecting one in every 1.5 million people per year [2]. GI complications are rarely manifested in this disease. Autoimmune dysregulation of the GI tract is considered a potential cause. The literature review primarily identifies GI manifestations in the pediatric population [3]. We present a case of an adult patient newly diagnosed with NMDA encephalitis with intestinal pseudo-obstruction likely due to autoimmune enteropathy.

## Case presentation

A 38-year-old African American male with recently diagnosed convulsive seizure and brief psychotic disorder was admitted after three weeks of confusion, hallucinations, and bizarre behavior. The patient had been recently discharged four weeks before for an acute psychotic episode of unknown etiology; epilepsy was diagnosed during that antecedent admission. The family did not recognize any prior seizure or psychiatric disorder and stated that the patient had been in good health with no previous known medical history. Since his discharge, the patient has been compliant with his medication: levetiracetam 500 mg twice daily, lorazepam 1 mg three times daily, and risperidone 2 mg twice daily.

Vitals were normal on the initial presentation. Admission laboratory workup was significant for mild leukocytosis (white blood count of 10.9 x103/UL) and elevated alanine transaminase (84 U/L) and aspartate aminotransferase (85 U/L). Head CT scan was unremarkable, and so was magnetic resonance imaging (MRI) of the head obtained on day five of the admission. The sepsis workup was unremarkable with negative blood culture. Lumbar puncture was done with findings of cloudy serosanguinous fluid, elevated glucose (101), protein (126), and red blood cell count (4970). CSF culture (bacterial and fungal) and virological studies (Epstein-Barr virus, herpes simplex virus, varicella-zoster virus, *Cryptococcus*, and enterovirus) were unremarkable. An immunological study of the CSF revealed NMDAR antibodies. The patient was then diagnosed with acute psychosis due to NMDAR encephalitis based on CSF studies. On day two of admission, the patient became catatonic. Both high-dose benzodiazepines (lorazepam 3 mg four times daily) and steroids (intravenous (IV) hydrocortisone 200 mg every four hours) were started. His catatonic symptoms were slowly improving, but the patient remained confused and had frequent episodes of hallucinations.

**Figure 1:**
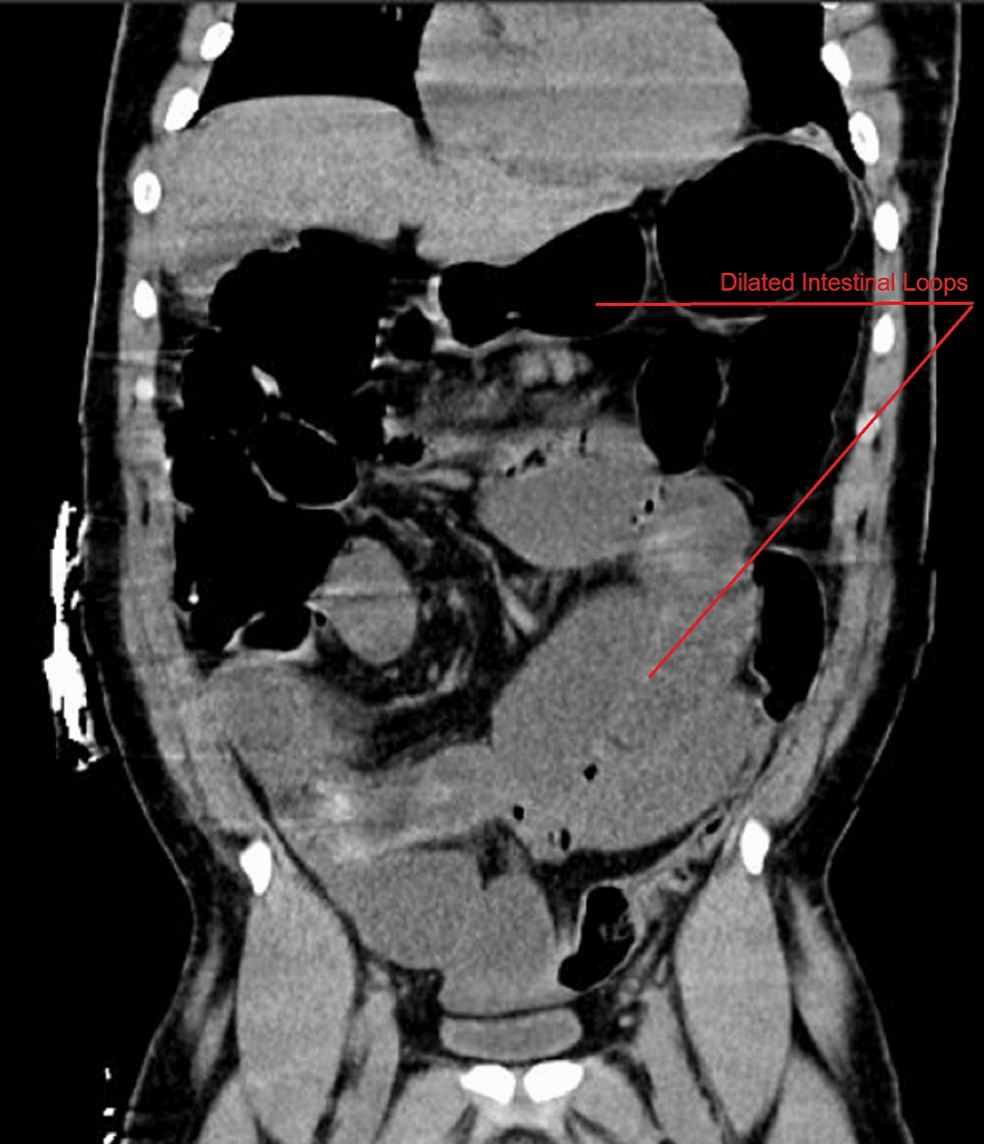
Abdominal CT scan with contrast showing distal small intestine obstruction with the swirling of mesenteric vessels.

On day seven, the patient developed abdominal tenderness with mild distension; a nasogastric (NG) tube was placed and drained 900 ccs of bilious and sanguineous fluid, concerning for GI bleeding. An abdominal X-ray revealed distension of the small bowel with concern for obstruction and ischemic bowel as lactic acidosis was found in laboratory workup. CT of the abdomen with contrast (Figure 1) showed diffuse moderate to pronounced dilated small intestine swirling the mesenteric vessels, concerning for intestinal vascular compromise.

**Figure 2:**
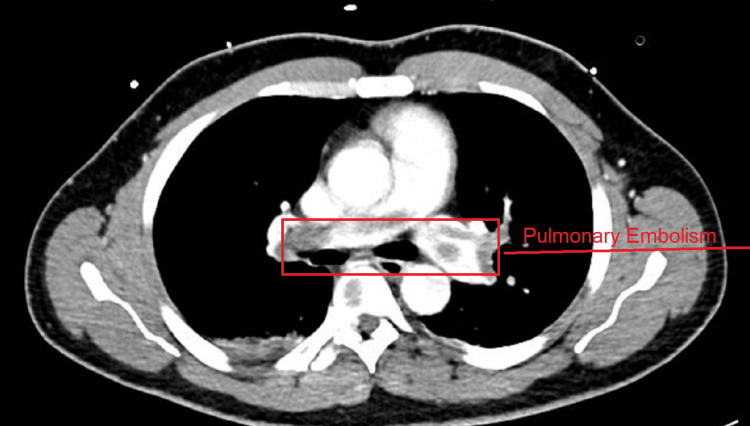
CT of the thorax with contrast showing massive pulmonary embolism within the left and right pulmonary arteries.

The patient also became tachypneic and hypoxic, requiring 6 L of oxygen with a venti-mask. CT of the chest, abdomen, and pelvis with contrast (Figure 2) found saddle pulmonary embolism (PE) extending to the right and left pulmonary artery with right heart strain.

**Figure 3:**
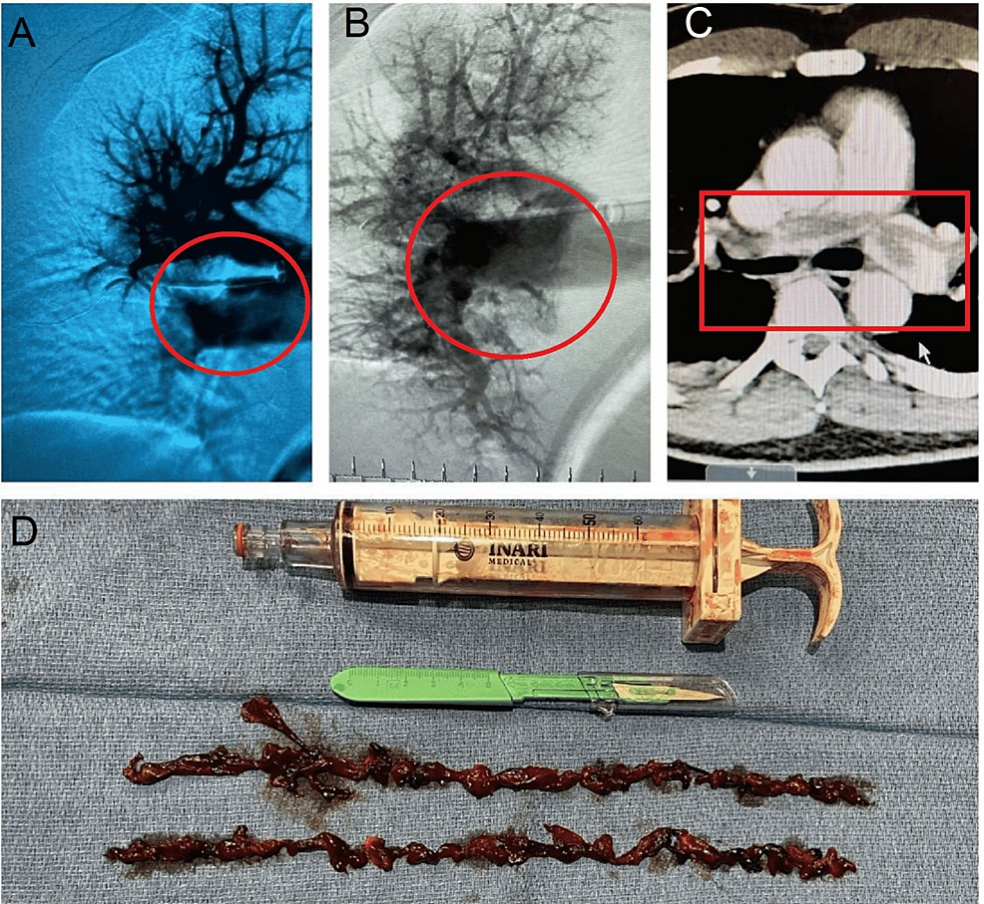
A-B: Extensive pulmonary embolism extended from the main pulmonary arteries into the lobar, segmental, and subsegmental branches in the upper and middle lobes, with lesser extend in the middle lobe. C: Massive pulmonary embolism with saddle emboli through the right and left main pulmonary arteries with right heart strain. D: Successful mechanical thrombectomy with FlowTriever catheter of the right main pulmonary artery, right truncus pulmonary artery, right middle lobe pulmonary artery, right interlobar pulmonary artery, right lower pulmonary lobe artery, left main coronary artery, and main pulmonary artery.

He was taken for emergent explorative laparotomy by the surgical team; cardiology was consulted for management of PE and advised emergent catheter-directed thrombectomy (Figure 3) with flow-retriever catheter before exploratory laparotomy. Neither necrotic bowel nor any evidence of perforation or volvulus was noted during the laparotomy; however, the small bowel and the colon were reported to be significantly dilated, hyperemic, and engorged with blood without any evidence of ischemic bowel. The surgical team proceeded with temporary partial closure with an abdominal wound vacuum and planned re-exploration. The heparin IV drip was started for PE with clearance from surgery. Abdominal re-exploration was done, and once again, no evidence of ischemic bowel was seen.

The patient’s course in the hospital was prolonged and complicated, requiring a multidisciplinary approach. Despite negative blood and intraoperative bowel fluid cultures during his admission, the patient developed septic shock requiring vasopressor support. The patient was then started on broad-spectrum antibiotics (vancomycin and cefepime) for a 14-day course after which his fever and leukocytosis resolved. Unfortunately, the patient could not be weaned off the mechanical ventilation required initially for the exploratory laparotomy. Fortunately, however, the patient’s neurological status improved once with a five-day course of intravenous immunoglobulin (IVIG) and high-dose steroids, with IV hydrocortisone 200 mg every four hours for anti-NMDA receptor encephalitis. Due to the patient’s multiple failed attempts on ventilator weaning trials, the patient was taken to the OR for tracheostomy on day 23 and gastrojejunostomy on day 29. Status post gastrojejunostomy tube placement, heparin drip was stopped, and the patient was started on enoxaparin instead as no further intervention was expected.

After one month of inpatient rehabilitation, the patient was stable and recovered functional capacity to be safely discharged to a skilled nursing facility for further care.

## Discussion

We report a case of a 38-year-old male with anti-NMDA encephalitis who was found to have small bowel distention without obstruction or ischemia and GI bleeding during his hospital stay. Later, surgical exploration revealed dilated small bowel without any source of obstruction or vascular compromise. Autoimmune enteropathy is a rare complication of anti-NMDA encephalitis stemming from dysregulation of the immune system. The systematic review by Giné-Servén et al. reports that 34.8% of adult patients with autoimmune encephalitis suffer from a motility disorder [4]. Yuan et al. performed a retrospective observational study on 143 patients with anti-NMDA receptor encephalitis and found the most associated findings to be seizures, psychiatric complications, pulmonary infections, and GI disorders. A total of 49% of patients experienced GI symptoms [5]. NMDAR are ligand-gated cation channels consisting of two subunits, NR1 and NR2, which bind glycine and glutamate, respectively. Overactivity of these receptors can present with symptoms of epilepsy, dementia, and stroke, whereas hypoactivity can give schizophrenic symptoms [6].

Autonomic dysfunction is the primary driving force behind GI symptoms in anti-NMDA encephalitis. Other common symptoms include sinus tachycardia, bradycardia, hypersalivation, central hypopnea, hypotension, and hyperhidrosis. Pathogenesis of autonomic dysfunction is via damage of autonomic nerve centers. Anti-NMDA encephalitis involves nerve centers in the limbic system associated with respiratory function, inotropic and chronotropic, GI motility, and other critical visceral activities. It is also common to be the originating region of epileptic activity [7]. Similar symptoms were seen in our patient who came with psychosis, despite being diagnosed with a recent new-onset seizure disorder, and during the hospital stay, he started having abdominal distention with concerns of obstruction. Another exciting finding present in our patient was the elevation of liver enzymes, which can also be seen with autonomic dysfunction of the liver. The pathogenesis behind this is damage to the ventromedial hypothalamus, leading to changes in liver cells [8]. Yan et al. showed that patients with autonomic dysfunction are more prone to intubation, mechanical ventilation, and intensive care unit admission [7].

GI symptoms with psychiatric diseases have always attracted neuroscientists. The gut-brain axis and role of gut microbiota have been previously studied as psychopathology in several psychiatric conditions, including schizophrenia [9]. A study of schizophrenia patients revealed significantly increased levels of pro-inflammatory markers, including interleukin 1, 6, and B in the patient’s GI tract. Inflammatory markers and the gut microbiome can alter the immune system and affect brain cognition in schizophrenia. Gut microbial dysbiosis plays a critical role in neuropsychiatric diseases and is also associated with chronic GI symptoms [10]. A study published in 2017 mentioned that probiotics improve the cortical neuronal response to NMDAR and improve cognitive flexibility [11]. This proves that dysregulation of NMDAR affects the gut-brain axis in multiple psychiatric conditions and likely neuropsychic disorders like anti-NMDA encephalitis.

This case is unique as anti-NMDA encephalitis rarely presents with GI pseudo-obstruction and PE. A literature review showed a case of anti-NMDA encephalitis with PE in a pediatric patient [12]. No specific pathogenesis has been studied for this hypercoagulability previously, but it is believed that a prolonged stay and IVIG treatment could propagate it [13]. NMDARs are present throughout the body, even in vasculature such as the pulmonary arteries. Nassar et al. mentioned the mechanism of tissue plasminogen activator (tPA) may act through NMDARs in the management of PE and that NMDAR antagonists can cause contractility of the vessel [14]. Although the mechanism of tPA or itself having antibodies against NMDARs causing PE is not established. More studies are essential for a more conclusive understanding of this disease and its pathophysiology.

## Conclusions

We highlight an exceptional and rare case of anti-NMDA encephalitis complicated by GI pseudo-obstruction and PE. Autonomic dysfunction is the main culprit for GI symptoms. The gut-brain axis as a pathophysiology is well established in other psychiatric diseases like schizophrenia and can have chronic GI symptoms. Physicians should keep in mind the gut-brain axis and autonomic effects on gut receptors of any patient presenting with psychosis and seizure disorder to provide timely care and improve morbidity and mortality in this patient population.
